# Optimization of Electrodialysis for Ammonium Removal From NH_4_Cl‐Doped Groundwater Samples Using the Response Surface Method

**DOI:** 10.1002/open.202400163

**Published:** 2024-10-23

**Authors:** Mohamed Hazra, Fatima Zahra Addar, Mustapha Tahaikt, Azzedine Elmidaoui, Mohamed Taky, Sakina Belhamidi

**Affiliations:** ^1^ Chemistry Superior School of Technology Ibn Tofail University P.O. Box 1246 Kenitra Morocco; ^2^ Chemistry Laboratory of Advanced Materials and Processes Engineering Faculty of Sciences Ibn Tofail University P.O. Box 1246 Kenitra Morocco

**Keywords:** Electrodialysis, Environmental chemistry, Optimization, Removal ammonium, Water chemistry

## Abstract

This study aims to optimize ammonium removal from NH_4_Cl‐enriched groundwater at different concentrations using an electrodialysis (ED) process. A customized design (CD) based on response surface methodology (RSM) was employed to develop predictive models and improve the performance of the demineralization system. Ion removal efficiency was evaluated in 32 unique experimental configurations, taking into account variations in three input parameters: voltage (A), initial ammonium concentration (B) and demineralization rate (C). These parameters were selected for their impact on two response variables: electric conductivity (Y_1_) and final ammonium concentration (Y_2_). An in‐depth analysis of variance (ANOVA) was performed to examine the variables and their interactions. The results indicated that Y_1_ was significantly influenced by C, while Y_2_ was influenced by B. In addition, the predictive models demonstrated strong correlations, with a coefficient of determination (R^2^) greater than 0.88 for both response variables. The RSM approach applied to optimize the parameters studied identified the following optimum values: 14.17 V for A, 1 mg/L for B and 70 % for C, giving Y_1_ of 215.377 μS/cm and Y_2_ of 0.279 mg/L.

## Graphical abstract

Response Surface Methodology (RSM) has proven its effectiveness in optimizing three key electrodialysis operational parameters for ammonium removal from groundwater in Kenitra, Morocco. The optimized parameters include a voltage of 14.17 V, an initial ammonium concentration of 1 mg/L and a demineralization rate of 70 %. These parameters resulted in an electric conductivity of 215.377 μS/cm and a final ammonium concentration of 0.279 mg/L.

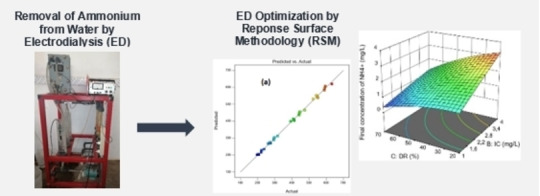

## Introduction

Agriculture plays a key role in the Moroccan economy, accounting for approximately 14 % of GDP in 2022, according to data from the Ministry of Agriculture, Fisheries, Rural Development, Water and Forests.^[1]^ Despite initiatives such as the “Green Morocco Plan”, launched in April 2008 for the horizon 2020, and the “Green Generation”, inaugurated on February 13, 2020 for the horizon 2030, which aim to mitigate the climatic impact of this sector, its development remains heavily dependent on the overexploitation of underground water resources. This overexploitation results in water shortages in certain regions of the country^[2]^ and pollution of surrounding water resources due to the excessive use of nitrogenous chemical fertilizers.^[3]^

Ammonium pollution in water has been reported in several studies on water quality in different parts of the country, with levels far exceeding the maximum permissible standards of 0.5 mg/L,[^4^, ^5^, ^6^] reaching up to 12 mg/L in some groundwater sources.^[7]^ The main sources of ammonium pollution include contaminated land, agricultural nitrogen fertilizers, animal manure, livestock farming, and industrial waste residues.^[8]^ This pollution can also lead to nitrate contamination, as nitrifying bacteria transform ammonium into nitrite and then nitrate. The adverse effects of drinking nitrate‐rich water are well‐documented, with the most frequently cited being methemoglobinemia (blue baby syndrome) and potential links to certain forms of cancer.[^9^, ^10^, ^11^] Additionally, high concentrations of NH_4_^+^ in waterways disrupt natural nutrient cycles and promote eutrophication.

It is therefore crucial to remove ammonium from groundwater before it is used and from wastewater before it is discharged into the environment to avoid contaminating natural water resources.^[12]^ Effective treatment methods are essential to guarantee optimal water quality and reduce the risk of eutrophication in aquatic ecosystems.

With this in mind, electrodialysis (ED) has been proposed as a technology to remove ammonium from groundwater originating from the city of Kenitra in Morocco.^[13]^ ED is an electromembrane method that effectively removes ionic compounds from water using an arrangement of ion exchange membranes (IEMs) that separate ions from aqueous solutions under the influence of an electric current.[^14^, ^15^, ^16^, ^17^] Compared to other membrane technologies such as reverse osmosis (RO), commonly used for water demineralization, ED is recommended due to its high water recovery, long membrane lifespan, low sensitivity to changes in feed water quality, and lower specific energy consumption (SEC) compared to the high‐pressure RO process in brackish water demineralization.[^14^, ^18^, ^19^] Furthermore, some studies suggest that ED is even more energy‐efficient when treating water with a salinity of less than 5000 mg/L.[^20^, ^21^]

The problem of ammonium pollution in groundwater is a delicate one. There is extensive discussion in the scientific literature about the removal or recovery of ammonium from wastewater using electrodialysis (ED), but to our knowledge, no work has been done on the removal of ammonium from groundwater by ED. This is probably due to the relatively lower concentration of ammonium in groundwater compared with wastewater, making ED treatment a complex process. Additionally, ED operation is strongly influenced by many factors. Voltage, flow rate, temperature, feed water composition, duration, removal rate, and ion exchange membranes (IEMs) characteristics have the greatest impact on the efficiency and economics of the ED system, while pH, though less influential, remains significant.[^14^, ^19^, ^22^, ^23^] With this in mind, we felt it was important to use tools capable of predicting ED performance as a function of the most significant parameters, in order to reduce the considerable number of experiments and contribute to a better understanding of the treatment process.

In recent years, the application of mathematical and statistical models to predict and improve the performance of chemical processes has developed. Among these techniques, Response Surface Methodology (RSM) stands out as one of the most widely used and effective approaches in chemistry. RSM relies on statistical and mathematical methods to compare theoretical predictions with experimental data, enabling the optimization of dependent variables as a function of various independent variables.[^24^, ^25^]

In principle, RSM involves fitting a polynomial equation to experimental data in order to statistically describe the behavior of a given data set. Among the types of RSM, the customized model based on historical data occupies a special place. This approach relies on the use of past data to develop a model specifically tailored to the unique characteristics of the experiment or process at hand. Unlike the standard approach, this method creates a model that takes into account the specific variables influencing the chemical process in question. Consequently, customization based on historical data emerges as a powerful strategy for optimizing complex chemical processes.[^26^, ^27^]

This customized approach based on historical data not only yields more accurate results but also provides a more comprehensive consideration of the factors that can influence a chemical process. By harnessing the wealth of historical data available, researchers and engineers can better understand the nuances of the process at hand. This deeper understanding enables them to make informed decisions and adjustments that lead to improved process efficiency and product quality. Furthermore, the customization based on historical data offers the advantage of adaptability. It allows for real‐time adjustments and fine‐tuning as new data becomes available during the ongoing chemical process. This adaptability is especially crucial in dynamic environments where external factors or unexpected variations can impact the process.[^28^, ^29^]

In our previous study,^[13]^ ammonium was successfully removed by ED from groundwater in the city of Kenitra, Morocco. Building on this basis, the present work focuses on the modeling and optimization of the ED treatment process. To this end, we chose to use RSM based on customized historical design (CD). This approach enabled us to assess the effects of three key factors – voltage (A), initial ammonium concentration (B), and demineralization rate (C) – on two crucial response parameters: electric conductivity (Y_1_) and final ammonium concentration (Y_2_). The aim was to determine the optimum values of these operational parameters in order to minimize these two critical process responses. For this purpose, experimental data were collected and a second‐order polynomial model was fitted. This model was then subjected to an analysis of variance (ANOVA) for rigorous statistical validation. Response surface curves were then generated, providing a powerful visual representation of the relationship between input variables and responses, complementing the regression equations.

## Materials and Methods

### Groundwater in the City of Kenitra – Morocco

Electrodialysis (ED) operations were carried out on groundwater samples from the city of Kenitra, Morocco, to which NH_4_Cl was added to obtain different NH_4_^+^ concentrations. The ionic composition of the groundwater studied without the addition of NH_4_Cl and the Moroccan standards (NM 03.7.001)^[30]^ for each parameter are presented in Table 1. These characteristics correspond to the average values observed throughout the study period.

**Table 1:** Average composition of groundwater studied.

Parameters	Values	Moroccan Standards (NM 03.7.001)
EC* (μS/cm), at 20 °C	720	2700
T (°C)	20.36	–
pH	7.75	6.5–8.5
NH_4_^+^ (mg/L)	0.37	0.5
SO_4_^2−^ (mg/L)	22.62	400
NO_3_^−^ (mg/L)	15	50
P‐Alkalinity (meq/L)	0	–
T‐Alkalinity (meq/L)	4.53	–
Hardness (meq/L)	5.88	–
Ca^2+^ (mg/L)	97.79	–
Mg^2+^ (mg/L)	12.07	–
HCO_3_^−^ (mg/L)	276.21	–
K^+^ (mg/L)	10.64	–
Na^+^ (mg/L)	106.22	200

*Electric conductivity.

Moroccan regulations (NM 03.7.001) do not impose standards for alkalinity and hardness, including Ca^2+^, Mg^2+^ and HCO_3_^−^. For K^+^, NM 03.7.001 does not specify a maximum limit. However, international recommendations suggest that the concentration of potassium in drinking water should be less than 12 mg/L for reasons of organoleptic quality and safety.

Ammonium ions (NH_4_^+^), the first mineral residues resulting from the decomposition of nitrogenous organic matter by ammonification, are frequently introduced into the soil via livestock effluents. Due to their positive charge, these ions are easily retained by the soil‘s clay‐humus complex, which explains their relatively low concentration in groundwater,^[31]^ so their presence in quantities above standardized limits is an indicator of groundwater pollution.

In groundwater samples from the city of Kenitra, NH_4_^+^ concentrations are generally below the 0.5 mg/l standard. So, to obtain NH_4_^+^ concentrations above this regulatory limit for our study, NH_4_Cl was used to create different NH_4_^+^ concentrations exceeding the prescribed limits.

### Electrodialysis Experiments and Membranes Selection

ED experiments were carried out on a TS‐2‐10 pilot plant supplied by Eurodia Co. (France), and the ion exchange membranes used were manufactured by Tokuyama Co. (Japan), type AXE for anion exchange membranes (AEMs) and CMX for cation exchange membranes (CEMs). AXE and CMX membranes are non‐selective, allowing the passage of all ions, from the most to the least charged.^[13]^ The characteristics of the TS‐2‐10 pilot and the properties of the membranes are similar to those reported in previous works.[^13^, ^17^, ^32^]

**Figure 1:**
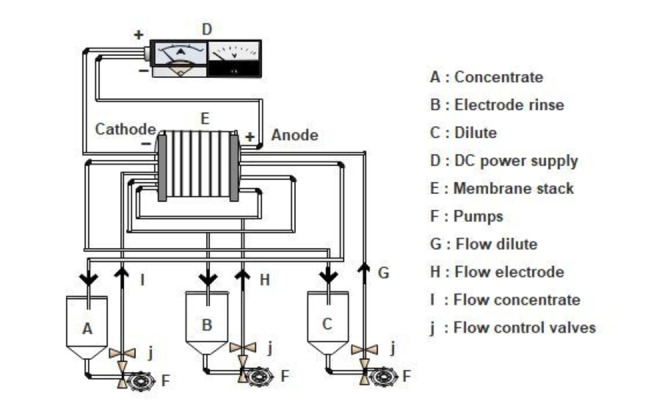
TS‐2‐10 electrodialyzer schematic diagram.

Three circuits make up the TS‐2‐10 pilot: one concentrate circuit, one dilute circuit, and one electrode circuit. Each compartment (dilute, concentrate, electrodes) was supplied with a volume of 2 L. A schematic representation of the TS‐2‐10 pilot plant is shown in Figure 1.

The dilute and concentrate compartments received the same raw solutions, while the electrode compartment was supplied with water enriched with NaCl to maintain a higher electric conductivity (EC) than the dilute and concentrate compartments, thereby minimizing energy consumption. Four NH_4_^+^ concentrations were studied (1, 2, 3, and 4 mg/L) at a constant flow rate of 180 L/h and two variable voltages of 10 V and 15 V. The flow rate choice was based on the results of Elmidaoui et al.^[23]^ who reported an optimal flow rate of 180 L/h in their study of nitrate removal using the same electrodialyzer. The removal time was recorded for each concentration at different demineralization rates (20, 40, 60, and 70 %). A maximum demineralization rate (DR) of 70 % was set to ensure sufficient mineralization in the dilute stream, thus avoiding various phenomena that limit ED, including proton leakage through the IEMs, concentration polarization, and current limitation. The electrodialyzer was washed with 0.1 N hydrochloric acid after each demineralization cycle to prevent salt precipitation and ensure that the initial membrane conditions were restored for the entire study.

### Analysis of Solutions

Water samples were periodically collected from the dilute stream for each NH_4_^+^ concentration (1, 2, 3 and 4 mg/L) studied at the two voltage settings (10 and 15 V) and subsequently subjected to physico‐chemical analysis.^[12]^ Spectrometric measurements were performed to quantify alkali ions K^+^ and Na^+^ using an industrial flame emission spectrometer (model PFP7 JENWAY). NH_4_^+^ and SO_4_^2−^ ions were quantified by spectrometry using a UV/VIS spectrometer (model UV1600) at wavelengths of 655 nm and 650 nm, respectively. NO_3_^−^ ions were determined potentiometry using selective electrodes and an ion meter (model Hach SensionTM^+^ MM340). pH measurements were conducted using a Jenway 3510 pH meter electrode, and electric conductivity was evaluated using a WTW Inolab Level 1 conductivity cell. Other parameters (HCO_3_^−^, total alkalinity, hardness, Ca^2+^, Mg^2+^) were determined using standard methods.

The demineralisation rate (DR) was calculated from the electric conductivity (EC) in the feed (E_f_) and the EC in the dilute (E_d_) according to equation 1:

(1) $$DR\;\left(\%\right)=\left(\frac{E_{f}-E_{d}}{E_{f}}\right)\times 100$$

### RSM Statistical Analysis Method

CD approach implemented in this project constitutes a subset of RSM and is employed to establish the relationship between operational variables and system responses. To this end, we used previous experimental data from 32 experimental runs. These data include three key input variables: voltage (A), initial ammonium concentration (“IC”) (B) and DR (C). In addition, they include two key responses: EC (Y_1_) and final ammonium concentration (Y_2_). Details of these data are presented in Table 2.

**Table 2:** Independent variables, Their Levels, and Symbols for CD.

Factor	Name	Units	Minimum	Maximum	Mean
A	Voltage	V	10	15	12.50
B	IC^[a]^	mg/L	1	4	2.50
C	DR^[b]^	%	20	70	47.50

[a] Initial ammonium concentration. [b] Demineralization rate.

To analyze and model the performance governing the ED process, we imported these data into Design Expert software (version 13), integrating ANOVA to establish interactions between process variables and assess the reliability of the mathematical model. This approach enabled us to describe the system‘s behavior in precise detail, based on past experience. It is thus an effective approach for optimizing the process using historical information, while avoiding repeating costly and time‐consuming experimental cycles. We utilized a quadratic polynomial model, as described in equation (2). This mathematical equation effectively represents the relationship between the input variables (A, B, C) and the response variables (Y_1_, Y_2_) in a quadratic format.

(2) $$Y=b_{0}+\sum_{i=1}^{n}b_{i}x_{i}+\sum_{i=1}^{n}b_{ii}x_{i}^{2}+\sum_{i=1}^{n-1}\sum_{j=i+1}^{n}b_{ij}x_{i}x_{j}$$

The dependent variables and the constant coefficients of this model have been comprehensively described in a previous study.^[25]^ The design matrix obtained after the application of CD is mentioned in Table 3.

**Table 3:** Matrix CD.

Experiences	Factor A (V)	Factor B (mg/L)	Factor C (%)	Response Y_1_ (μS/cm)	Response Y_2_ (mg/L)
1	10	1	20	523.2	1.26
2	10	1	40	392.4	1.2
3	10	1	60	261.6	1.01
4	10	1	70	196.2	0.48
5	10	2	20	551.2	1.94
6	10	2	40	413.4	1.72
7	10	2	60	275.6	0.96
8	10	2	70	206.7	0.94
9	10	3	20	548.8	2.78
10	10	3	40	411.6	2.14
11	10	3	60	274.4	1.33
12	10	3	70	205.8	0.75
13	10	4	20	543.2	3.32
14	10	4	40	407.4	2.89
15	10	4	60	271.6	1.91
16	10	4	70	203.7	1.2
17	15	1	20	588.8	1.15
18	15	1	40	441.6	0.77
19	15	1	60	294.4	0.68
20	15	1	70	220.8	0.36
21	15	2	20	590.4	1.36
22	15	2	40	442.8	1.18
23	15	2	60	295.2	0.78
24	15	2	70	221.4	0.28
25	15	3	20	594.4	3.43
26	15	3	40	445.8	2.73
27	15	3	60	297.2	1.54
28	15	3	70	222.9	0.94
29	15	4	20	632.8	2.76
30	15	4	40	474.6	2.14
31	15	4	60	316.4	2.02
32	15	4	70	237.3	1.32

## Results and Discussion

**Figure 2:**
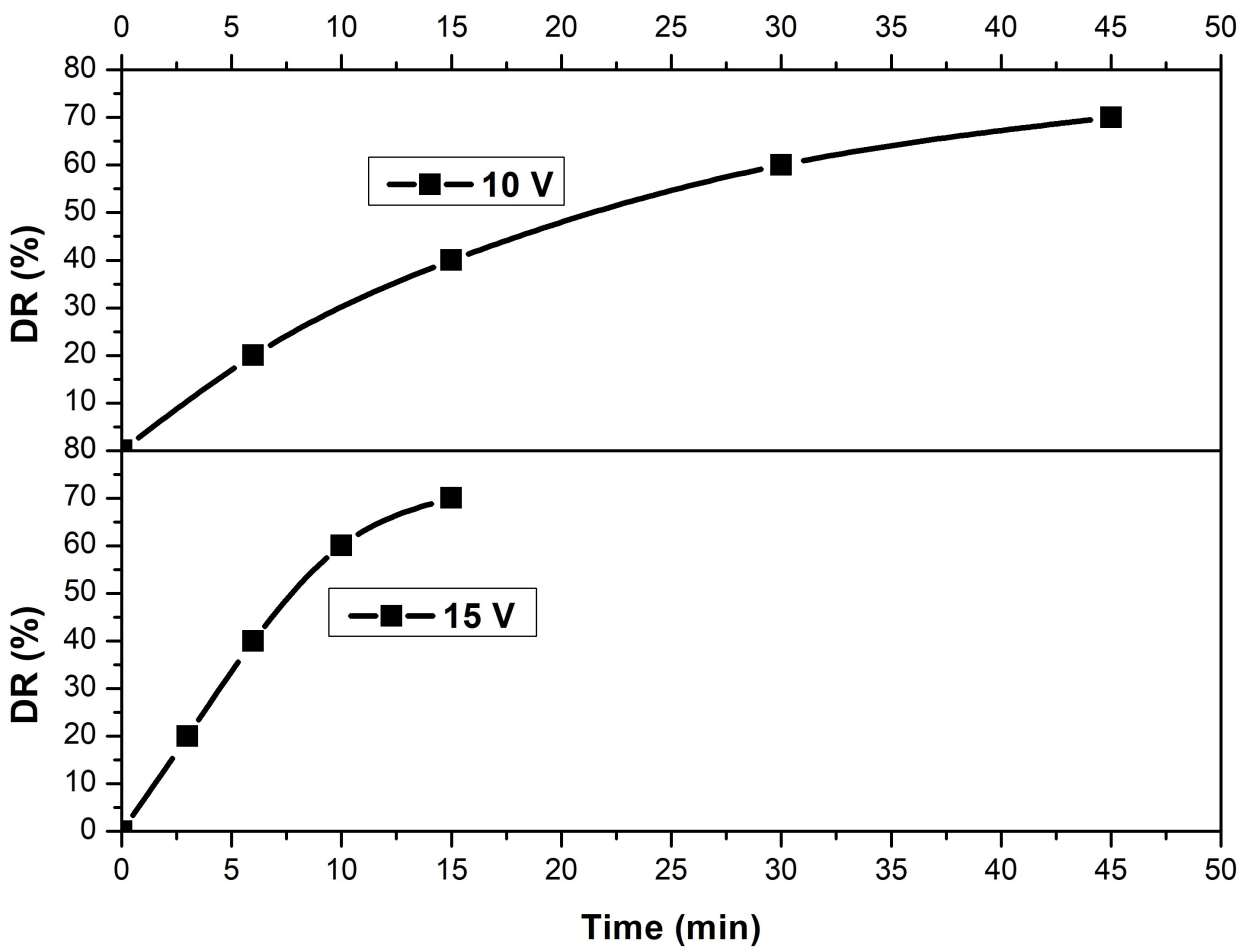
Demineralization rate variation as a function of time for 10 V and 15 V.

First, we monitored the demineralization rates (20, 40, 60 and 70 %) of each solution as a function of time for each voltage (10 and 15 V). Figure 2 is a time mean of the four concentrations studied for each voltage.

We observe that the rate of demineralization (DR) increases with time for both voltages. However, at a voltage of 10 V, it takes 45 minutes to reach a DR of 70 %, whereas at 15 V, it takes only 15 minutes to reach the same DR. Treatment time and voltage are two parameters that significantly influence the efficiency of electrodialysis (ED), as the amount of ions transported from the dilute to the concentrated compartment is governed by electrical charge, which is the product of current intensity (a consequence of voltage) and time.^[18]^ Research by Karimi et al.^[22]^ highlights the critical importance of voltage in ED systems and the production of high‐quality demineralized water. According to the Nernst‐Planck equation, voltage influences ion transport by diffusion and electromigration. Higher voltage favors electromigration of ions, thus improving their elimination. However, this effect varies with ion type, due to different diffusivities and ion‐ion interactions. At higher voltages, the percentage of ion removal approaches 100 %. Banasiak et al.^[33]^ have reported that the time required to reduce the NaCl concentration to 500 mg/L for drinking water decreases as the applied voltage increases. With an initial NaCl concentration of 5000 mg/L, the times were 70, 50 and 27 minutes at 9, 12 and 18 V, respectively. A similar trend was observed with an initial NaCl concentration of 10 000 mg/L, with times of 73 and 37 minutes at 12 and 18 V, respectively.

Next, the results of ANOVA for the two models, Y_1_ and Y_2_, used in this study are presented in Tables 4 and 5. The correlation coefficient R^2^ indicates the extent to which the models explain the variation in responses.

**Table 4:** ANOVA for the response Y_1_.

Source	Sum of Squares	Df	Mean Square	F‐value	p‐value	R^2^
Model	6.19E+05	8	77320.2	1254.17	<0.0001	0.9977
A‐Voltage	13385.63	1	13385.63	217.12	<0.0001	
B‐IC^[a]^	1742.03	1	1742.03	28.26	<0.0001	
C–DR^[b]^	6.02E+05	1	6.02E+05	9765.12	<0.0001	
AB	264,71	1	264.71	4.29	0.0496	
AC	1659.38	1	1659.38	26.92	<0.0001	
BC	215.95	1	215.95	3.5	0.074	
A^2^	0	0				
B^2^	2.2	1	2.2	0.0358	0.8517	
C^2^	0	1	0	0	1.0000	
Residual	1417.96	23	61.65			
Cor Total	6.20E+05	31				

[a] Initial ammonium concentration. [b] Demineralization rate.

**Table 5:** ANOVA for the response Y_2_.

Source	Sum of Squares	Df	Mean Square	F‐value	p‐value	R^2^
Model	20.38	8	2.55	22.58	<0.0001	0.887
A‐Voltage	0.1877	1	0.1877	1.66	0.2099	
B‐IC^[a]^	9.88	1	9.88	87.54	<0.0001	
C–DR^[b]^	9.69	1	9.69	85.87	<0.0001	
AB	0.0693	1	0.0693	0.6143	0.4412	
AC	0.0123	1	0.0123	0.1086	0.7447	
BC	1.01	1	1.01	8.97	0.0065	
A^2^	0	0				
B^2^	0.0034	1	0.0034	0.0302	0.8636	
C^2^	0.2611	1	0.2611	2.31	0.1419	
Residual	2.59	23	0.1128			
Cor Total	22.98	31				

[a] Initial ammonium concentration. [b] Demineralization rate.

For the Y_1_ model, the R^2^ is 0.99, meaning that the model explains around 99.7 % of the variation observed in the data. The ANOVA shows that the overall model is significant, with a high Fisher coefficient value (F‐value) of 1254.17 and a very low probability value (p‐value) (<0.0001). This indicates that the model is statistically significant in explaining variations in Y_1_. Among the individual factors, A, B, C and AC have significant effects on Y_1_.

The Y_2_ model displays a high R^2^, at 0.887, meaning that it explains around 88.7 % of the variability present in the Y_2_ data. Furthermore, the ANOVA reveals a significant overall model, characterized by a high F‐value of 22.58 and a very low p‐value (<0.0001). In terms of individual factors, B and C stand out as having significant effects on Y_2_.

The two equations (3) and (4) presented express mathematical models describing the relationship between input variables (A, B, C) and system responses (Y_1_ and Y_2_). Each of these equations represents a quadratic polynomial relationship between the input variables and the system responses.

(3) $$Y_{1}=392.65+20.62A+9.98B-178.63C+3.86AB-9.37AC-4.54BC+0.5906B^{2}$$

The variables A, B and their interaction AB have significant positive effects on the value of Y_1_, contributing to its improvement. Conversely, variables C, AC and BC have marked negative effects, reducing the value of Y_1_, with the effect of C being the most pronounced among them. Variables B^2^ have a negligible impact on Y_1_, having little influence on response.

(4) $$Y_{2}=1.75-0.0772A+0.7516B-0.7166C+0.0624AB{+0.0255AC-\;0.3106BC-\;0.0232B}^{2}-{0.2173C}^{2}$$

Variables B, C and their interaction BC exert significant negative effects on the Y_2_ response. These effects are particularly strong in the model as a whole, and outweigh the other factors in terms of significance. Moreover, the effects of B and C have similar coefficients, meaning that they contribute proportionally to the overall negative impact on Y_2_. The other factors have negligible effects in comparison.

**Figure 3:**
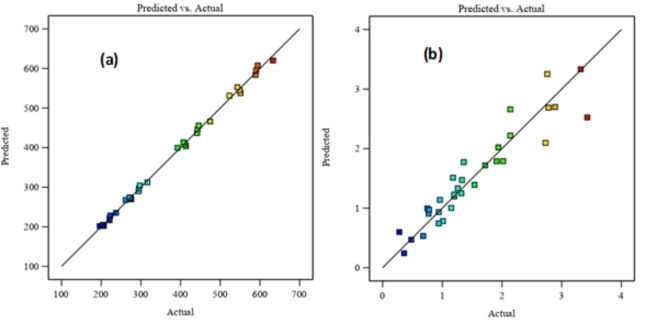
Experimental values of Y_1_ (a) and Y_2_ (b) versus predicted data.

Figure 3 compare the experimental values of two responses (Y_1_ and Y_2_) to the predicted data calculated.

It shows curves representing both actual and predictions data for both responses. These graphs illustrate excellent agreement between the values predicted by the models and the experimental data observed in the study area. Moreover, the points cluster closely along the diagonal line, indicating that the errors follow a normal distribution. This convincingly confirms that the regression models (Y_1_ and Y_2_) correspond exceptionally well to the actual values, as shown by correlation coefficients in excess of 0.88 for both models.

### Response Surface (3D)

**Figure 4:**
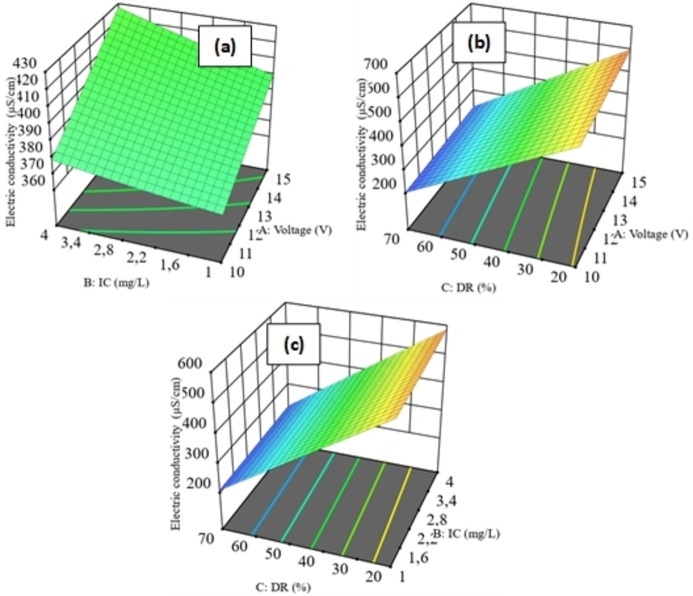
3D response surfaces of the effect of the interaction between the three parameters (A, B and C) on Y_1_. (a): 3D contour plot of the effect of A and B on Y_1_. (b): 3D contour plot of the effect of A and C on Y_1_. (c): 3D contour plot of the effect of B and C on Y_1_.

**Figure 5:**
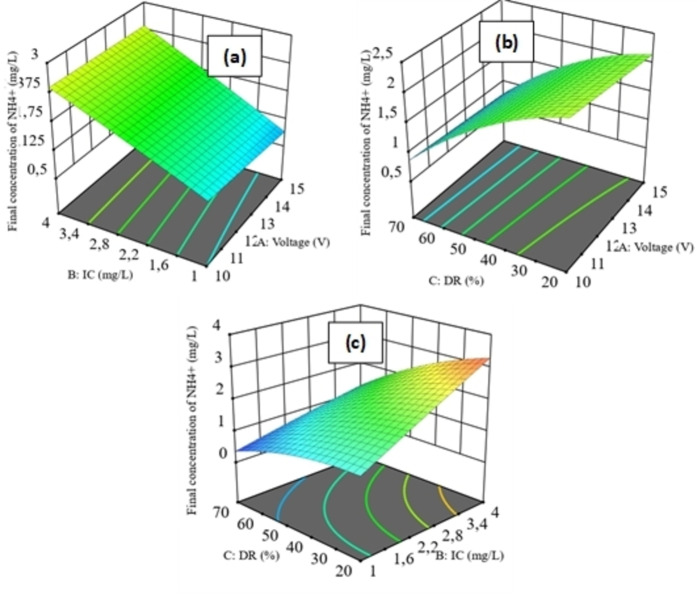
3D response surfaces of the effect of the interaction between the three parameters (A, B and C) on Y_2_. (a): 3D contour plot of the effect of A and B on Y_2_. (b): 3D contour plot of the effect of A and C on Y_2_. (c): 3D contour plot of the effect of B and C on Y_2_.

The 3D response surface plots (Figures 4 and 5) show the effect of the interaction between the three variables: voltage (A), IC (B) and DR (C) on the responses: EC (Y_1_) and final ammonium concentration (Y_2_).

The 3D response surface plots (Figure 4 (a) to (c)) show the effects of interactions between variables A, B and C on the Y_1_ response. Observations on all three plots suggest that the main effect is based on the C variable. As C increases, Y_1_ decreases. The EC of a solution is affected by the equivalent concentration and equivalent conductivity of the ions present in the solution.^[34]^ As the ion concentration decreases, the EC decreases and, consequently, the rate of demineralization increases. Furthermore, the interaction between A and C has a significant effect on Y_1_. Indeed, ED efficiency depends on the applied voltage;^[35]^ as voltage increases, the DR rises. Y_1_ decreases, reaching minimum values of 200 μS/cm. Similarly, the interaction between B and C has an effect on Y_1_. Y_1_ decreases as C increases and B decreases. Conversely, the interaction between A and B has no effect on Y_1_. The composition of the feed water plays an important role in the efficiency of ED operation;^[36]^ although A and B increase, the decrease in Y_1_ only occurs if the composition of the feed water decreases. Aliascari and Schäfer^[37]^ reported that, although voltage is used to control the ED process, water quality characteristics can affect contaminant removal. In addition, the ionic characteristics of contaminants determine their removal by ED, and higher feed concentrations lead to higher final contaminant concentrations in dilute water.

The 3D response surface diagrams Figure 5 show the effects of interactions between variables A, B and C on the Y_2_ response.

The observations in Figure 5 (a) to (c) clearly show that the predominant element, which has a direct impact on Y_2_, is variable B, particularly in the upper limits. Y_2_ decreases until it becomes almost zero. Additionally, the interaction between B and C proved to be a significant factor on Y_2_. In other words, when C increases and B decreases, Y_2_ decreases. Indeed, during ED operation, NH_4_^+^ finds itself in ionic competition with other cations present in the water, due to their high concentration, higher valence (Ca^2+^ and Mg^2+^), ionic mobility, hydration energy and size, geometric structure, etc.[^29^, ^38^, ^39^] As the concentration of the majority of cations decreases, C creases and Y_2_ decreases more readily. However, voltage seems to have minimal influence on Y_2_. It is known that at higher applied voltages, the demineralization is close to 100 %. According to several studies, not all ions are affected in the same way by voltage, and it has been observed that different ionic responses result from different ionic diffusivities and ion‐ion interactions in solution and in IEMs used.[^23^, ^35^, ^40^]

### RSM Optimization

**Figure 6:**
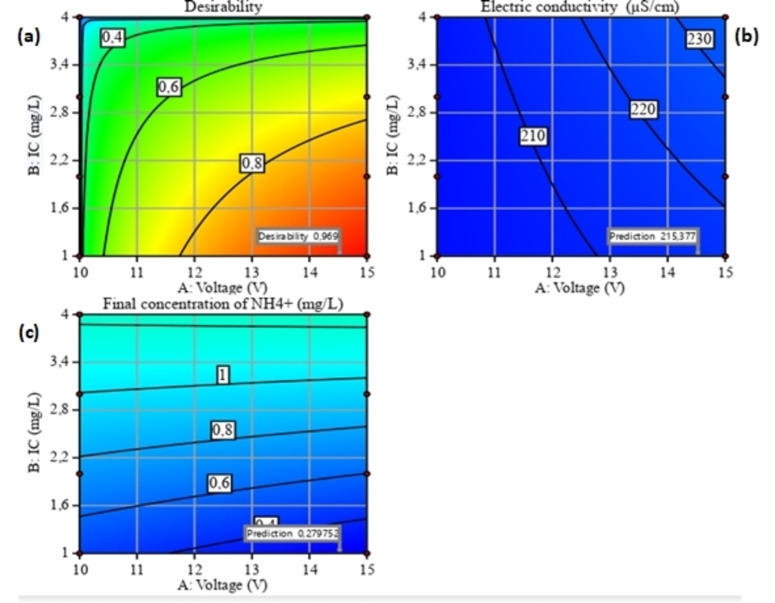
Optimal conditions for minimizing the two responses (Y_1_‐Y_2_) for the interaction between two parameters (A and B).

**Figure 7:**
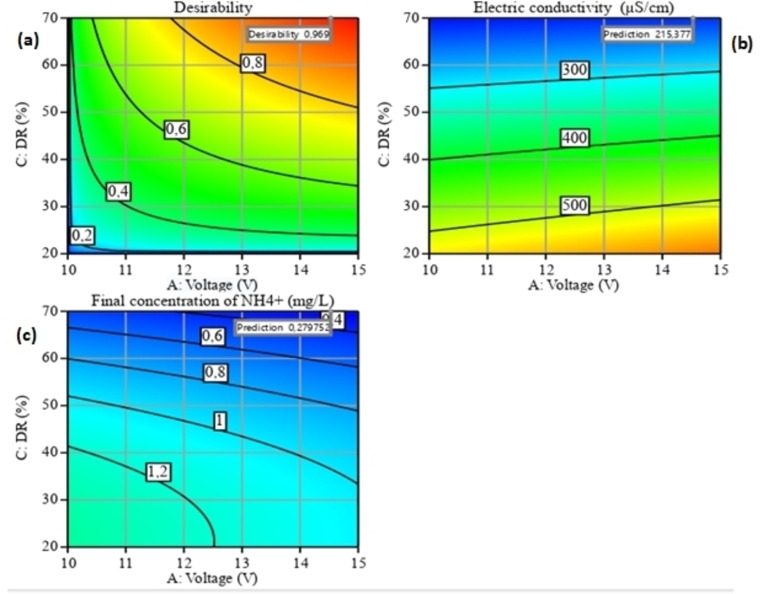
Optimal conditions for minimizing the two responses (Y_1_‐Y_2_) for the interaction between two parameters (A and C).

**Figure 8:**
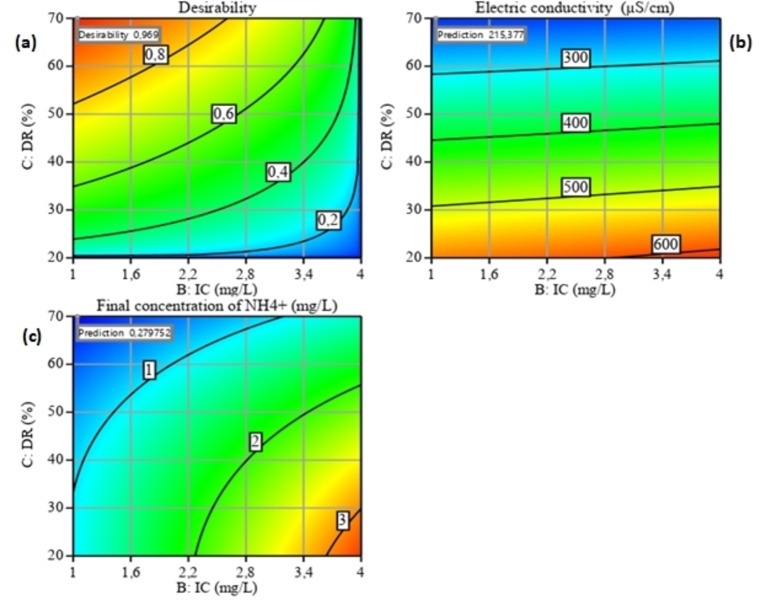
Optimal conditions for minimizing the two responses (Y_1_‐Y_2_) for the interaction between two parameters (B and C).

Optimization of the ion removal process parameters (Figure 6, 7 and 8) by ED was carried out using Design Expert software. The objective of the optimization process was set to minimize EC (Y_1_) and the final ammonium concentration (Y_2_) within the experimental range of the independent variables studied. The optimization solution is generally selected on the basis of greatest desirability or proximity to the unit; in general, the red zone is the area where maximum removal efficiency can be found.^[41]^

Optimum conditions for A, B and C were obtained for both responses at values of (14.17 V, 1 mg/L, 70 %), respectively. Under these optimum conditions, Y_1_ and Y_2_ were achieved respectively (215.377 μS/cm – 0.279 mg/L). These predictive results are in good agreement with the experimental results.

However, improving other ED operating parameters can increase demineralization efficiency. For example, Nguyen et al.^[18]^ used RSM to optimize three key parameters – voltage, feed water volume and treatment time – to simulate the demineralization process of water initially containing 5693 mg/L total dissolved solids (TDS). Their findings indicate that feed water volume exerts the most significant influence on demineralization efficiency, followed by treatment time and voltage. Another study by Bachiri et al.^[42]^ showed that time significantly affected hardness removal, and that the interaction between tension and initial hardness had an impact on final hardness. Furthermore, Gardeshi et al.^[43]^ observed in their research that flow rate and time played an important role in chloride removal.

In addition, the application of the RSM method has proven to be an effective approach for the systematic optimization of response variables, enabling resource and cost savings by reducing energy consumption, maximizing membrane utilization and improving overall operational efficiency.^[42]^

## Conclusions

In this work, an electrodialysis process was used to remove ammonium from real solutions consisting of groundwater from the city of Kenitra in Morocco, spiked with NH_4_Cl at concentrations ranging from 1 to 4 mg/L NH_4_^+^. The model used to predict and optimize the electrodialysis process led to the following conclusions:

- The demineralization rate is the most important factor affecting the electric conductivity of the produced water, and its interaction with the other parameters (voltage and initial ammonium concentration) also influences the electric conductivity of the produced water.
- Initial ammonium concentration is the most important factor affecting final ammonium concentration. Its interaction with the demineralization rate influences the final ammonium concentration.
- Feed water composition plays an important role in ammonium removal.
- The optimum conditions obtained with the RSM method for voltage, initial ammonium concentration and demineralization rate are 14.17 V, 1 mg/L and 70 % respectively for an electric conductivity and a final ammonium concentration of 215.377 μS/cm and 0.279 mg/L respectively.
- The experimental operating conditions resulting from the modelling led to the finding that electrodialysis could be a safe and reliable solution for removing ammonium from groundwater.

This study will contribute to research into the depollution of natural waters by cleaner methods such as electrodialysis.

## Conflict of Interests

The authors declare no conflict of interest.

1

## Data Availability

The data that support the findings of this study are available from the corresponding author upon reasonable request.
